# Use of Telemedicine to Expedite and Expand Care During COVID-19

**DOI:** 10.5811/westjem.2021.4.51317

**Published:** 2021-08-19

**Authors:** Meeta Shah, Keya Patel, Daniel Popa, Anthony Perry, Shayna Adams, Braden Hexom, Carter Neugarten, Michael Gottlieb

**Affiliations:** * Rush University Medical Center, Department of Emergency Medicine, Chicago, Illinois; † Rush University Medical Center, Department of Internal Medicine, Chicago, Illinois

## Abstract

**Introduction:**

The novel coronavirus disease 2019 (COVID-19) created challenges with access to care including increased burden on healthcare systems and potential exposure risks for vulnerable patients. To address these needs, Rush University Medical Center created a virtual, urgent care program specifically designed to address these challenges during the COVID-19 pandemic.

**Methods:**

This was a retrospective study analyzing adult patients with COVID-19-related telemedicine visits performed between March 1–June 30, 2020. COVID-19-related telemedicine visits refer to those who used the “Concern for Coronavirus” module. We assessed the total number of telemedicine visits using this module, percentage with a subsequent emergency department (ED) visit within seven days, and outcomes (ie, hospitalization status, intubation, and death) of patients who presented to the ED for evaluation. Data are presented using descriptive statistics.

**Results:**

A total of 2,974 adult patients accessed the program via the COVID-19 module over the four-month period. Of those, 142 patients (4.8%) had an ED visit within seven days. Only 14 patients (0.5%) required admission. One patient was intubated, and there were no deaths among the telemedicine population.

**Conclusion:**

The data suggests that telemedicine may be a safe and effective way to screen and treat patients with possible COVID-19, while reducing potential burdens on EDs.

## INTRODUCTION

At the time of submission, there were 17 million cases of coronavirus disease 2019 (COVID-19)^1^ in the United States alone.^2^ This has led to increased emergency department (ED) visits and hospital admissions.^3^ Telemedicine has emerged as one avenue to increase capacity for medical care during this pandemic. Previously, data has been inconsistent on the clinical and cost effectiveness of telemedicine.^4^,^5^ In 2018, 83% of surveyed healthcare system executives reported plans to invest in telehealth; however, most cited that their major barriers were reimbursement and licensure issues.^6^ For states without reimbursement parity, telehealth services could not compare to reimbursement from in-person care. Illinois did not have significant telehealth coverage prior to 2019. Rush University Medical Center sought to increase telehealth access with a particular focus on COVID-19 upon implementation of state and federal parity allowances during the pandemic.^7^,^8^

In this study we sought to determine specific utilization of our virtual urgent care platform Rush University Medical Center, for COVID-19-related presentations during a four-month period. Additionally, we sought to describe subsequent outcomes with regard to ED visits within seven days, including admissions, intubations, and death among this population.

## METHODS

### Study Design and Participants

This was a retrospective, observational study analyzing all Rush University Medical Center “Concern for Coronavirus” video visits performed at Rush University Medical Center. Rush University Medical Center is a quaternary-care healthcare system in the Midwest, which includes three hospitals comprising one academic medical center with an annual ED volume of 72,000 patients/year and two community hospitals with a combined annual ED volume of 130,000 patients/year. We included all adult patients (defined as age ≥ 18 years) who used a “Concern for Coronavirus” video visit with a licensed provider between March 1–June 30, 2020. The start date was selected to coincide with when the first patient in our region presented.^9^ While the Rush University Medical Center telemedicine program has been present since August 19, 2019, it was significantly expanded on March 5, 2020, to accommodate the increasing number of potential COVID-19 patients that could present to the ED or clinics with their concerns. This study was deemed exempt by the Rush University Medical Center Institutional Review Board.

### Data Collection

We obtained data from an analytics dashboard created by the institution’s knowledge management team that extracts discrete data from our electronic health record (Epic Systems Corporation, Verona, WI). We also collected summative response data from satisfaction surveys distributed to patients routinely after their video visits and determined the number of detractors (score 0–6), passives (score 7 or 8), and promoters (9 or 10). We determined the Net Promoter Score (Satmetrix, Inc., Redwood, CA; Bain & Company, Inc. and Fred Reichheld) by subtracting the percentage total of detractors from the percentage total of promoters.

We extracted data from all telemedicine visits using the “Concern for Coronavirus” module. We subsequently extracted data on patients who had an ED visit within seven days of their video visit. Chart reviews were performed in accordance with best practice guidelines.^10^

We trained two investigators on data extraction and provided a list of variables with a codebook of definitions. One investigator extracted all data into a pre-designed and pre-piloted worksheet. A second investigator independently abstracted 15% of the charts to assess accuracy. The kappa between chart abstractors was 0.89 (95% confidence interval [CI], 0.74, 1.00). Any discrepancies were resolved by a third abstractor. We abstracted the following data: age; gender; race; smoking status; comorbidities; COVID-19 testing; date of telemedicine visit; data of ED visit; whether the patients were hospitalized; hospitalization status (eg, observation, general medical floor, intensive care unit); whether they were intubated during the hospitalization; and whether they died during the hospitalization.

### Statistical Analyses

We presented continuous data as mean with standard deviation (SD). Categorical data were presented as number and percentage. We analyzed all data with Microsoft Excel version 16.35 (Microsoft Corporation, Redmond, WA). Cohen’s kappa was calculated for the dual extraction using SPSS version 26 (IBM Corp., Armonk, NY).

## RESULTS

A total of 2,974 adult patients accessed the Rush University Medical Center telemedicine platform via the “Concern for Coronavirus” module. Of these, 142 patients (4.8%) had an ED visit within seven days and 14 patients (0.5%) required admission. One patient was intubated, and there were no deaths. Table 1 provides a summary of the basic demographics for the patients.

Of those who completed a telemedicine visit, 149 (4.2%) completed a post-visit survey (Table 2). The mean scoring on a 10-point Net Promoter question was 9.6/10.0 (SD: 1.1) demonstrating a Net Promoter Score of 89.9%, suggesting very high levels of patient satisfaction. Additionally, 89.9% felt their care was equal to or better than in-person care. Of importance, a substantial number of patients would have alternatively sought in-person care.

## DISCUSSION

Telemedicine has arisen as an additional modality to expand care during the COVID-19 pandemic. While the role of telemedicine continues to expand within medical care, the COVID-19 pandemic provided a unique situation to assess this expansion. Our study demonstrated that telemedicine was able to scale operations quickly to and provide care to a substantial number of patients with a low rate of total ED presentations. As most primary care offices in our system had more limited access during this time, these represent potential ED visits that were successfully managed using telemedicine with only 142 ED presentations. In fact, nearly 35% of surveyed patients reported they would have come to the ED. Ultimately, only 0.5% of all COVID-19-related telemedicine patients were subsequently hospitalized after their telemedicine visit. While it cannot be confirmed, this suggests that the telemedicine visits were able to address the patient’s COVID-19-related concerns while potentially reducing the burden and potential exposure to ED providers.

Many of the survey participants also stated that their care was equal to or better than in-person care. This is consistent with other studies of telemedical care demonstrating high patient satisfaction. A recent Press-Ganey study of over 3.5 million telemedicine patients found that virtual visits had similar patient experience ratings to in-person visits.^11^ Another study conducted prior to the COVID-19 pandemic found that telemedicine patients were less likely to return for additional evaluation and had higher Press-Ganey satisfaction scores when compared to fast-track ED patients.^12^

Post-pandemic, as the economy continues to struggle, there may be a rise in demand for more cost-efficient care via telemedicine. However, the most significant barriers to adopting a robust telemedicine program include training, resistance to change, cost, and reimbursement.^13^ The pandemic lowered many of these barriers. As clinics closed and access to in-person medical care became more difficult, the pandemic forced our hands in adapting newer modalities of healthcare that previously had been met with skepticism or resistance. Cost and reimbursement were also addressed by governing bodies who loosened regulations regarding payment for telemedicine services.

Of course, telemedicine has limitations in the care it can render. Outside of requesting patients to self-assess their vitals with any devices they may own at home (eg, thermometer, pulse-oximeter), there are restrictions on what patient data can be obtained. Future consideration in how to better distribute home monitoring devices to the general public could expand the usability of this technology.

As we move forward, new technology and infrastructure must be created to sustain the growth and expansion of telemedicine. This should be complemented with additional training for providers. Currently there are no guidelines for how telemedicine should be incorporated in resident education and little to no consistency in healthcare curriculum regarding telemedicine.^14^,^15^ Further study should assess how best to incorporate this method of healthcare delivery into residency training.

## LIMITATIONS

There are several important limitations to consider in this study. First, this was a retrospective study at a single healthcare system in a single region. Patients tended to be younger, and may not reflect outcomes at other healthcare institutions. Moreover, our response on post-visit surveys was low, and it is possible that satisfaction results may have differed if a larger portion had completed the survey. Finally, patients who used our telemedicine service may have subsequently sought care in external EDs; thus, our data may not have captured all associated ED visits, admissions, or deaths.

## CONCLUSION

Our study found that the implementation of telemedicine during COVID-19 was an effective means of care for patients concerned about coronavirus disease 2019.

## Supplementary Information



## Figures and Tables

**Table 1:** Demographics of patients who used telemedicine.

Demographic	Number (%)
Age	
18–44	2,068 (69.5%)
45–65	799 (26.9%)
> 65	107 (3.6%)
Gender	
Female	1,863 (62.6%)
Male	1,108 (37.3%)
Unknown	3 (0.1%)
Race	
White	1,198 (40.3%)
Black or African American	723 (24.3%)
Asian	121 (4.1%)
American Indian or Alaska Native	14 (0.5%)
Native Hawaiian or other Pacific Islander	7 (0.2%)
Other	571 (19.2%)
Unknown	163 (5.5%)
Smoking Status	
Never Smoker	1,563 (52.6%)
Former Smoker	302 (10.2%)
Current Smoker	166 (5.6%)
Unknown	943 (31.7%)
Presence of co-morbidities	
Distinct patients with asthma	524 (17.6%)
Distinct patients with COPD	26 (0.9%)
Distinct patients with Diabetes	359 (12.1%)
Distinct patients with hyperlipidemia	249 (8.4%)
Distinct patients with hypertension	501 (16.8%)
Distinct patients with CAD	60 (2.0%)
Distinct patients with CHF	33 (1.1%)
COVID-19 Evaluation	
Distinct patients with a COVID-19 test order	2,116 (71.1%)
Distinct patients with a COVID-19 positive result	652 (21.9%)
Distinct patients from all patients who used the “Concern for Coronavirus” module and were seen in the ED within 14 days of their video visit.	296 (10.0%)

*COPD*, chronic obstructive pulmonary disease; *CAD*, coronary artery disease; *CHF*, congestive heart failure; *COVID-19*, novel coronavirus disease 2019; *ED*, emergency department.

**Table 2:** Patient satisfaction with telemedicine visit.

Patient satisfaction survey	Numbers (%)
Alternate care	
Number who would have sought in-person visit with a doctor	38 (25.5%)
Number who would have sought care at another healthcare organization	9 (6.0%)
Number who would have sought care with another video visit vendor	10 (6.7%)
Number who wouldn’t have received care	20 (13.4%)
Number who would have gone to Minute Clinic (eg, CVS, Walgreens)	11 (7.4%)
Number who would have gone to (blinded for peer review) emergency department or walk-in clinic	52 (34.9%)
Unanswered	9 (6.0%)
Perception of care	
Number who felt care was equal to or better than in-person care	134 (89.9%)
Number who felt care was worse than in-person care	9 (6.0%)
Unanswered	6 (4.0%)
Satisfaction scores (0–10)	
Number detractors (score 0–6)	1 (0.7%)
Number passives (score 7, 8)	13 (8.7%)
Number promoters (9,10)	135 (90.6%)
