# Supplementary material for: Use of Telemedicine to Expedite and Expand Care During COVID-19
Source: West J Emerg Med. 2021 Aug 19;22(5):1028–31. doi: 10.5811/westjem.2021.4.51317 (PMC8463054; doi:10.5811/westjem.2021.4.51317)
Supplement: Supplementary file 1 [file wjem-22-1028-s001.docx]

**Supplemental File: Patient Survey**

On a scale from 0-10, how likely are you to recommend a video visit to a friend or colleague?

- Sliding scale bar ranging from “Not very likely” to “Very likely”

What is the primary reason for giving the score you chose?

- Open ended answer

Was the visit easy to complete?

- Yes
- No

Were you able to get your healthcare issue resolved without needing to seek additional care?

- Yes
- No

If you would have sought care elsewhere, how would you have received it?

- Minute Clinic (for example, CVS, Walgreens, etc.)
- Rush Emergency Department or Walk-In Clinic
- Another Healthcare Organization
- Another Video Visit Vendor
- An In-person visit with a doctor
- I wouldn’t have received care

How would you compare the care you received during the Virtual Visit to an in person visit?

- Better than in person
- Equal to in person
- Worse than in person
